# Upregulation of Low-Density Lipoprotein Receptor of the Steroidogenesis Pathway in the Cumulus Cells Is Associated with the Maturation of Oocytes and Achievement of Pregnancy

**DOI:** 10.3390/cells10092389

**Published:** 2021-09-11

**Authors:** Myung Joo Kim, Young Sang Kim, Yu Jin Kim, Hye Ran Lee, Kyoung Hee Choi, Eun A Park, Ki Ye Kang, Tae Ki Yoon, Sohyun Hwang, Jung Jae Ko, You Shin Kim, Jae Ho Lee

**Affiliations:** 1 CHA Fertility Center Seoul Station, Seoul 04637, Korea; splower2@cha.ac.kr (M.J.K.); p0892@chamc.co.kr (Y.S.K.); hrlee87@chamc.co.kr (H.R.L.); nankhee76@chamc.co.kr (K.H.C.); eapark9327@chamc.co.kr (E.A.P.); eladynaye@chamc.co.kr (K.Y.K.); tkyoon@chamc.co.kr (T.K.Y.); 2 Department of Obstetrics and Gynecology, CHA University School of Medicine, Seoul 04637, Korea; 3 Laboratory of Reproductive and Molecular Medicine, CHA Fertility Center Seoul Station, Seoul 04637, Korea; yj_kim@chamc.co.kr; 4 CHA Bundang Medical Center, CHA University, Seongnam 13496, Korea; blissfulwin@chamc.co.kr; 5 Department of Biomedical Science, College of Life Science, CHA University, Pocheon 11160, Korea

**Keywords:** cumulus cells, ageing, transcriptome, next-generation sequencing, steroidogenesis

## Abstract

The maturation of the oocyte is influenced by cumulus cells (CCs) and associated with pregnancy rate, whereas the influencing factors have not been completely elucidated in the CCs. In this study, we identified new regulators of CCs for high-quality oocytes and successful pregnancies during assisted reproductive techniques. CCs were collected from cumulus–oocyte complexes (COCs) in young (≤33 years old) and old (≥40 years old) women undergoing intracytoplasmic sperm injection (ICSI) procedures. We screened for factors differentially expressed between young vs. old CCs and pregnancy vs. non-pregnancy using whole mRNA-seq-next-generation sequencing (NGS). We characterized the transcriptome of the CCs to identify factors critical for achieving pregnancy in IVF cycles. Women in the young and old pregnancy groups exhibited the up- and downregulation of multiple genes compared with the non-pregnancy groups, revealing the differential regulation of several specific genes involved in ovarian steroidogenesis in CCs. It was shown that the low-density lipoprotein (LDL) receptor to the steroidogenesis pathway was upregulated in CCs with higher maturity rates of oocytes in the pregnancy group. In conclusion, a higher pregnancy rate is related to the signaling pathway of steroidogenesis by the LDL receptor in infertile women undergoing IVF procedures.

## 1. Introduction

In assisted reproductive technology (ART), age is a major determinant of successful pregnancy outcomes [1,2,3]. Currently, no curative treatment is available for infertility related to advanced age, which manifests as a reduced competence of oocytes and embryos in ART [1]. Follicular development is a highly complex process regulated by multiple factors in gonadotropin-independent and dependent phases [4,5]. Oocyte quality is associated with the functional competence of somatic cells in the ovary, including granulosa cells (GCs) and Cumulus cells (CCs) [6,7,8,9], which are responsible for nurturing oocyte growth, development, and the gradual acquisition of developmental competence [10]. Among women of advanced age undergoing in vitro fertilization (IVF), those with a history of successful live births may have different GC and CC responses during follicular development from those who have experienced failed pregnancies [11]. Oocytes from women over 40 years old have reduced developmental competence. To date, however, no critical factors have been identified that can mitigate the risk of not achieving pregnancy at advanced maternal age.

CCs have different roles and sustain the activities of different factors required for the production of competent oocytes depending on the phase of folliculogenesis [12,13]. The initial follicle development phase (pre-antral phase: primary and secondary follicle) is regulated by undifferentiated GCs. After antrum formation in the antral phase, the GCs differentiate into functionally distinct lineages: the mural GCs, which line the wall of the follicle and play key steroidogenic roles, and CCs, which form intimate associations with the oocyte. After antral follicular development, the oocyte gradually and sequentially acquires meiotic and developmental competence [7]. Several reports have suggested that the oocyte acquires the molecular and cytoplasmic machinery that fully support oocyte development during the antral phase of follicle development [14]. However, we still have only a poor understanding of the nature and diversity of the compounds that are transferred between CCs and the oocyte during antral development, and it remains unclear whether dynamic changes depend on aging [15]. Therefore, CCs sustain the activities of several factors for oocytes’ maturation and ovulation and determine the competence of embryo development for successful pregnancy [14]. Factors expressed by CCs are useful as non-invasive predictors of oocyte quality, embryo development, and clinical pregnancy ratios during IVF procedures [13,16,17].

Until now, several studies aimed at identifying the predictive biomarkers of oocyte and embryo competence have reported the RNA profiling of specific factors in human CCs using microarrays and next-generation sequencing (NGS) [16,18]. However, the extent of studies using NGS or microarray techniques is limited because they are restricted to studying oocyte quality, embryo development, or pregnancy-related predictive biomarkers as they relate to clinical outcomes [17,19,20,21,22,23]. One study using mRNA-seq NGS of CCs from women of advanced age reported that hypoxia is one factor involved in follicular senescence, but it does not identify pregnancy-related factors in aged CCs [24]. To our knowledge, there has been no study employing mRNA profiling in CCs to identify the factors that affect oocyte maturation and pregnancy outcomes of advanced age women. Women over 40 years of age only rarely have good quality oocytes or produce competent embryos. We hypothesized that CCs express factors that can influence the likelihood of a successful pregnancy in advanced age women.

Here, we focus on identifying factors that are differentially regulated between young and advanced age women and between those who have achieved successful pregnancies and those who have not (hereafter, referred to as pregnancy and non-pregnancy groups, respectively). We aimed to use NGS-based, whole-transcriptome databases to detect new regulators of ovulation in high-quality oocytes that are likely to increase the rate of successful pregnancies in advanced age IVF patients.

## 2. Materials and Methods

### 2.1. Collection of Human CCs during IVF 

This study was approved by the Institutional Research and Ethical Committees of CHA University (approval number: 1044308-201611-BR-027-04), Republic of Korea. All participating researchers underwent training and received a certificate for biomedical research with human materials. All women gave written informed consent to provide material for this study. All procedures followed the rules for studies with human-origin materials established by the IRB. Patients were divided into four groups according to age (“young” ≤ 33 years old vs “old” ≥ 40 years old) and pregnancy: (1) young pregnancy (Young P, *n* = 8); (2) young non-pregnancy (Young NP, *n* = 8); (3) old pregnancy (Old P, *n* = 8); and (4) old non-pregnancy (Old NP, *n* = 8). No personal information about study subjects was collected other than check-up age and category of infertility diagnosis. Patients participating in the study had been diagnosed with infertility due to male, tubal, or uterine factors. Women with infertility due to endocrine diseases, such as polycystic ovarian syndrome (PCOS) or women with genetic diseases were excluded. All patients underwent conventional controlled ovarian stimulation (COS) or minimal stimulation protocols with a Gonadotropin-releasing hormone (GnRH) antagonist protocol during the IVF procedure. When there was at least one or more 18-mm follicle, ovulation was triggered by recombinant human chorionic gonadotropin (hCG) injection and oocytes were retrieved 36 h later. After ovum pick-ups, we isolated CCs by denuding oocytes with 0.1% hyaluronidase (ORIGIO CooperSurgical, Måløv, Denmark) for ICSI. Isolated CCs were washed in PBS and stored at −80 °C until mRNA purification. Eight samples were collected for each group. All clinical data were collected using electronic medical records, including baseline characteristics (age, body mass index (BMI), infertility factors, previous IVF cycles, etc.) and basal hormone levels. Parameters related to IVF cycles were also investigated, including number of retrieved oocytes, and maturation, fertilization, and clinical pregnancy rates. A high-quality embryo at the cleavage stage was defined as a 4-cell embryo on day 2 and a 7- or 8-cell embryo on day 3, containing <20% anucleate fragments, and exhibiting no apparent morphological abnormalities. As for blastocyst-stage embryos, a good-quality embryo was defined according to the Gardner and Schoolcraft criteria [25]. 

### 2.2. mRNA Extraction and mRNA-seq NGS Analysis

mRNA was isolated from CCs from each group using the DynaBeads mRNA DIRECT Kit (Life Technologies, Oslo, Norway). mRNA was quantified using a NanoDrop ND-1000 spectrophotometer (Nyxor Biotech, Paris, France). Purified mRNA was subjected to mRNA-seq using a MiSeq sequencer, (MiSeq System, Illumina, San Diego, CA, USA), and sample sheets were prepared to provide run details. Bioinformatic analysis was performed on the whole-transcriptome raw data obtained from NGS.

### 2.3. Bioinformatics Analysis of mRNA-seq NGS Data 

Whole-transcriptome data were analyzed using the human genome sequence as reference (UCSC hg19, annotation RefSeq_2017_06_12). From the initial NGS data, 27,685 genes were identified under the 4 sample conditions using the stringTie-e option. The data were then normalized to fragments per kilobase of exon per million fragments mapped (FPKMs) using Cufflinks. To filter potentially significant gene expression, genes with more than one FPKM value of 0 were excluded. To clarify the relationship between samples, unsupervised hierarchical clustering analysis and multidimensional scaling (MDS) analysis were performed on the expression data for 17,317 genes; we then sought to confirm the presence of outlier samples and similar expression patterns between biological replicates.

Next, we performed differential expression gene (DEG) analysis. For this purpose, log2(FPKM + 1) values were calculated and normalized by the quantile method. Transcripts with fold-change values >2 with *p*-value ≤ 0.05 were included as DEGs in the subsequent analysis (total, 1366). To display DEG expression patterns, hierarchical clustering analysis was performed using complete linkage and Euclidean distance as a measure of similarity. The top 10 Gene Ontology terms, in ascending order of *p*-values, are shown. Next, we analyzed the list of top-ranking up- and downregulated genes and plotted each up and down gene cluster as a function of age and pregnancy outcome. 

Each group was investigated according to age and pregnancy competence. To identify key DEGs associated with pregnancy competence, we compared three pairs: (1) old pregnancy vs. old non-pregnancy; (2) young pregnancy vs. young non-pregnancy; and (3) pregnancy vs. non-pregnancy, regardless of age. In the first two comparisons, we used fold-change values (>2 or <0.5) to identify DEGs, as each group contained only one sample. In the final comparison, we analyzed the data using the t-test, and *p*-values were adjusted by the Benjamini–Hochberg procedure. However, because all adjusted *p*-values were statistically insignificant, we used *p* < 0.05 instead of adjusted *p* < 0.05 to identify DEGs.

### 2.4. Gene Ontology and Enrichment Analysis

DEG data were uploaded to the online Database for Annotation, Visualization, and Integrated Discovery (DAVID) program [26]. Functional categories were clustered using the functional annotation clustering tool, and representative Gene Ontology (GO) categories were identified. Databases of GO/canonical pathways and hallmark gene sets were used to infer significantly enriched terms in each gene set using Metascape (http://david.abcc.ncifcrf.gov/tools.jsp, v.6.8 web software). After GO analysis, Kyoto Encyclopedia of Genes and Genomes (KEGG) pathway analysis was performed to identify ovary specific signaling pathways in the gene enrichment data of DEGs with *p*-value < 0.05. The threshold was set as modified Fisher’s Exact *p*-value (EASE score) ≤ 0.05.

### 2.5. Validation Study of NGS Data by Reverse Transcriptome-qPCR

We performed a validation study by RT-qPCR to identify and confirm data for the young and old pregnancy groups. mRNA was purified using the DynaBeads mRNADIRECT Kit. Total RNA was reverse transcribed to cDNA using the AccuPower^®^ CycleScript RT PreMix (Bioneer, Daejeon, Korea) with poly-dT. qPCR was performed with AccuPower Taq PCR PreMix (Bioneer) in a spectrofluorometric thermal cycler (SimpliAmp Thermal Cycler; Thermo Fisher Scientific). Based on the KEGG signal pathways, we selected up- and downregulated target genes involved in ovarian steroidogenesis as a function of age and pregnancy outcome. Real-time qPCR was performed using the SsoAdvanced Universal SYBR Green Supermix (Bio-Rad, Hercules, CA, USA) with specific primers (Table S2) for the genes encoding low-density lipoprotein receptor (LDLR), steroidogenic acute regulatory protein (StAR), adenylate cyclase (ADCY), hydroxysteroid 17-beta dehydrogenase 1 (HSD17B1), and β-actin (used as a normalization control). The comparative threshold cycle (CT) method was used, and the level of PCR product for each gene was normalized against the corresponding level of the PCR product for β-actin. Samples were evaluated in triplicate. PCR products were electrophoresed on 2% agarose gels and visualized using SafeView (Applied Biological Materials, Richmond, Canada). Gels were imaged under UV illumination using a gel documentation system (WSE-6100 LuminoGraph; ATTO, Tokyo, Japan). Finally, the product bands were subjected to intensity ratio analysis using the ImageJ software.

### 2.6. Statistical Analyses

Statistical analyses were performed using SPSS 22 (SPSS Inc., Chicago, IL, USA). All data are expressed as means ± standard error of the mean (SEM) of triplicate measurements. Statistical analyses were carried out using t-tests with significance level set at * *p* < 0.05.

## 3. Results

### 3.1. Characteristics of Clinical Samples and Oocyte Profiling Depend on the Patients

**Scheme 1:**
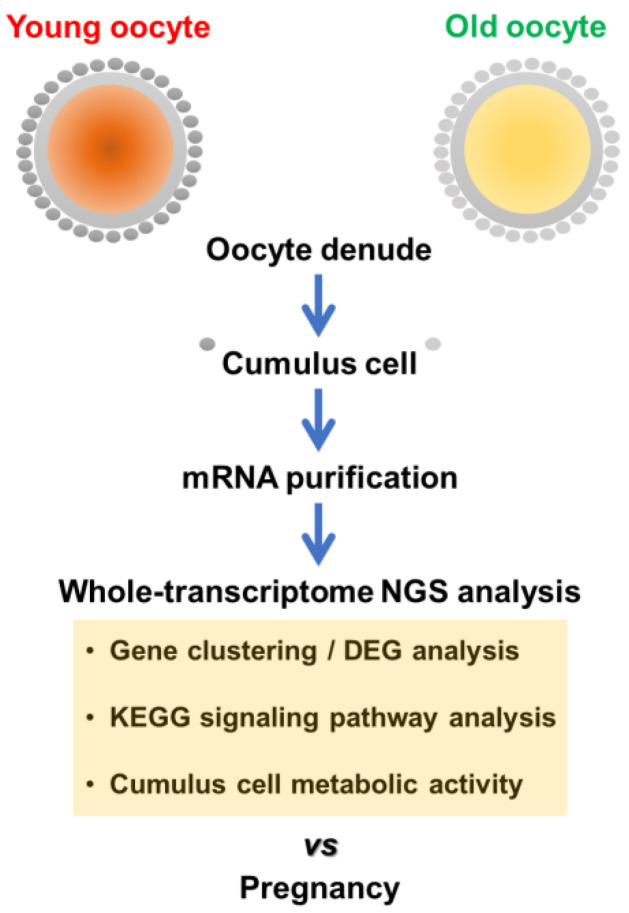
Design of sample groups for whole-transcriptome analysis of CCs.

Table 1 shows the characteristics of the samples used for NGS transcriptome data analysis. The collected CCs were classified as young (≤33 years old, *n* = 16) or old (≥40 years old, *n* = 16) and pregnancy or non-pregnancy. Age-related AMH was significantly reduced in the old group relative to the young group. However, the clinical outcomes did not significantly differ within each age group as a function of BMI, number of previous IVF cycles, or basal FSH level. All IVF patients were stimulated using GnRH antagonist protocols with no significant difference in the doses of gonadotropin administered. The oocyte properties such as the number of retrieved oocytes and the fertilization rate did not significantly differ among groups. However, the oocyte maturation rates were significantly different among groups. Based on the clinical outcomes, pregnancy was related to oocyte and embryo quality in both the young and old groups. The clinical pregnancy group had a significantly greater number of blastocyst formation rates and higher number of high-quality blastocysts than the non-pregnancy group. Hence, we performed whole-transcriptome analysis by NGS following Scheme 1 to identify differences in the mRNA expression profiles between the pregnancy and non-pregnancy group and between young and old patients.

**Figure 1:**
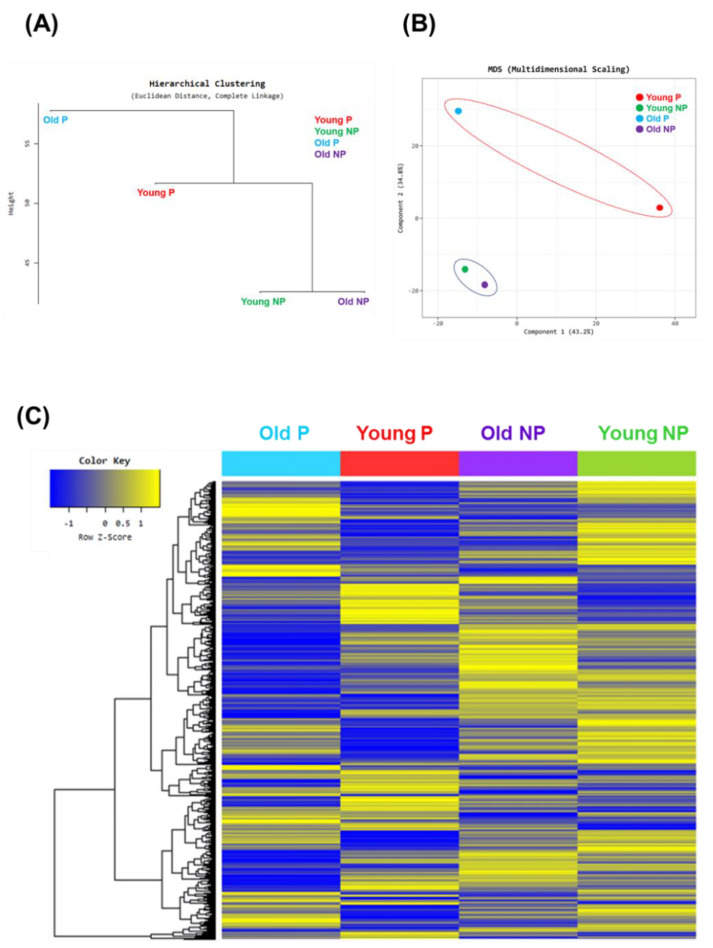
Graphs of Euclidean distance, multidimensional scaling analysis, and hierarchical clustering heatmap analysis. Hierarchical clustering by (**A**) Euclidean distance and (**B**) multidimensional scaling (MDS), comparing age and pregnancy: young pregnancy (Young P, red), young non-pregnancy (Young NP, green), old pregnancy (Old P, blue), old non-pregnancy (Old NP, purple). (**C**) Graph of hierarchical clustering heatmap indicating differentially up- and downregulated genes based on age and pregnancy outcome. Color intensity indicates gene expression, normalized using log2(FPKM) values. Yellow and blue indicate up- and downregulation, respectively.

Figure 1 shows a plot of hierarchical clustering (A) and multidimensional scaling analysis (B) of the data using normalized value (log2) based on age and pregnancy. Gene expression patterns in the pregnant group were significantly different than those in the non-pregnant group. Interestingly, the young and old non-pregnancy groups had similar gene profile ratios as evidenced by the similar Euclidean distances. In Figure 1C, the heat map of the hierarchical clustering graph clearly reveals similar z-scores within the non-pregnancy group (both young and old). In addition, the pregnancy groups had similarly high component expression levels, but the expression pattern differed between the young and old pregnancy groups. In addition, the expression pattern of the non-pregnancy group was similar between the young and old groups. In addition, the young and old pregnancy groups had stronger gene expression activity than the non-pregnancy groups. Interestingly, the young and old non-pregnancy groups had a lower gene expression profile than the young and old pregnancy groups (Figure 1C).

### 3.2. GO Analysis of Young vs Old and Pregnancy vs. Non-Pregnancy

**Figure 2:**
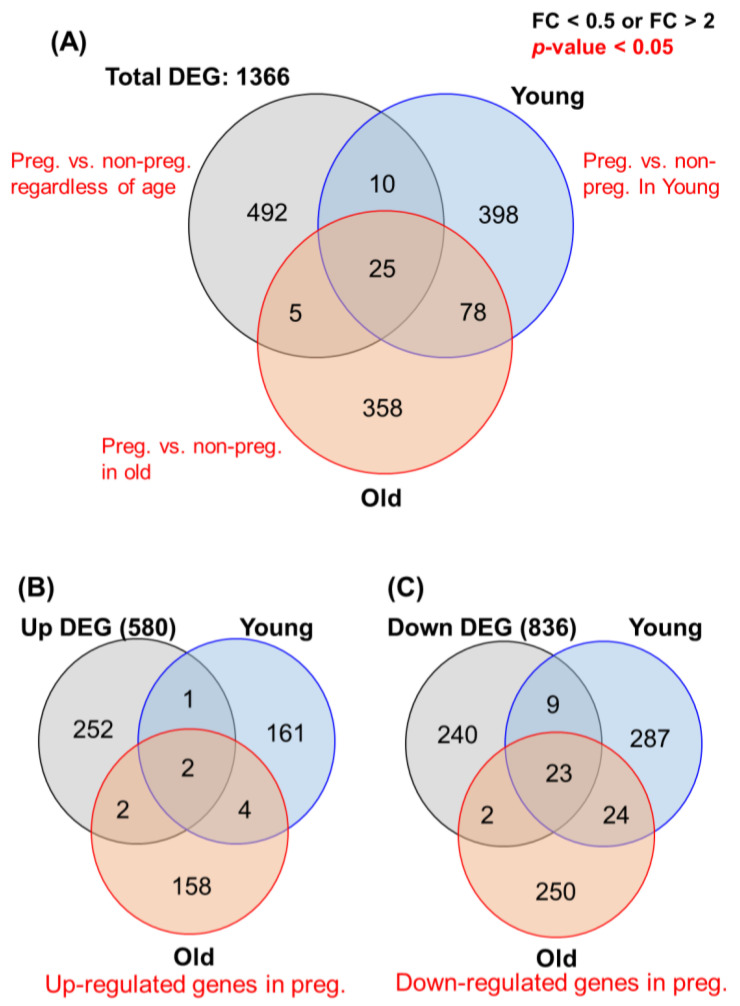
Total transcriptome profiling depending on sample criteria, based on DEG analysis of NGS data. (**A**) Venn diagram of significantly up- or downregulated genes (fold-change ≥ 2) in the four groups (young/old, pregnancy/non-pregnancy). A total of 1366 genes were included in the RNA-seq analysis: pregnancy vs. non-pregnancy regardless of age (492 genes), pregnancy vs. non-pregnancy in the young group (398 genes), and pregnancy vs. non-pregnancy in the old group (358 genes). (**B**) Venn diagram of 580 upregulated genes in the pregnancy group (young and old). (**C**) Diagram of 836 downregulated genes in the pregnancy group (young and old). Data are presented as means ± SEM of three replicates. Significant differences are indicated by asterisks (* *p* < 0.05).

Next, from a total of 17,317 genes, we identified those that exhibited a ≥2-fold change in expression (Figure 2A). Based on the DEG data, we identified 1366 genes that were ≥2-fold differentially regulated in the young or old group: 580 upregulated (Figure 2B) and 836 downregulated (*n* = 836) (Figure 2C). Table S1 lists the top 10 up- and downregulated genes of the young and old pregnancy groups. Most upregulated genes were associated with ovarian functions such as proliferation, differentiation, and steroidogenesis in CCs, e.g., integrin subunit alpha 2, small zinc finger protein 117, etc. By contrast, the roles of several genes have not been elucidated in the ovary, e.g., C1GALT1C1, SNORD150, etc. Generally, the GO functional analysis revealed the enrichment of the “biological process”, “cellular component”, and “molecular function” terms.

Both young and old pregnancy groups showed the upregulation of genes in the category “cellular component” such as: zinc finger protein, NME1-NME2 readthrough, and the HECT and RLD domain containing E3 ubiquitin protein ligase 2 pseudogene. The old pregnancy group exhibited elevated expressions of “single-organism process” genes such as NGFR. Among “cellular component” related genes, the young and old pregnancy groups showed significantly high expression of “cell part” and “cell-related” gene processes. Among molecular function genes, both young and old pregnancy groups showed significantly high expression of cell–cell adhesion related genes. Therefore, we performed a key signal pathway analysis of the functional network of each gene in young and old pregnancy patients.

### 3.3. Identification of Key Signal Map by KEGG Pathway Analysis

**Figure 3:**
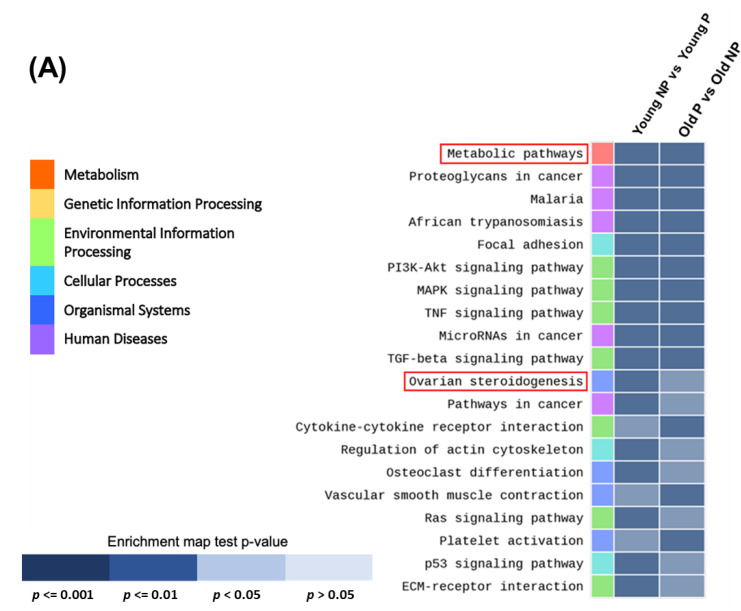

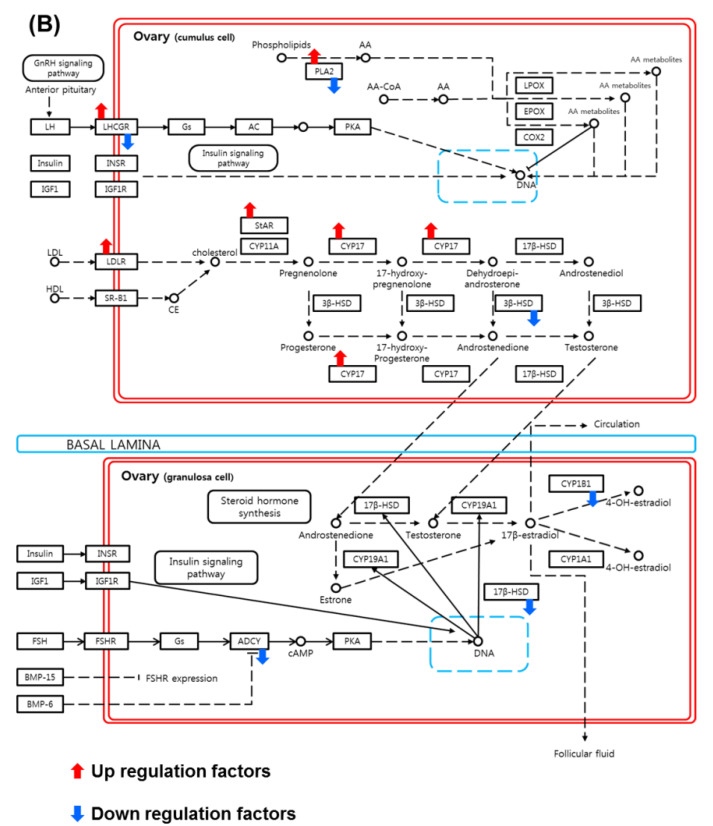
Functional annotation enrichment analysis of the whole-transcriptomes profile using Database for Annotation, Visualization and Integrated Discovery (DAVID). (**A**) Genes were categorized by gene set enrichment analysis according to age and pregnancy. Enrichment map test *p*-values, *p* < 0.001 to >0.05. Data are presented as means ± SEM of three replicates. Significant differences are indicated by asterisks (* *p* < 0.05). (**B**) Schematic of cumulus cell signaling pathway involving up- and downregulated genes, based on the ovarian steroidogenesis KEGG pathway map from the gene set enrichment analysis.

In both the real and simulated data of the young vs. old and pregnancy vs. non-pregnancy groups, we assessed the impact of the normalization methods using the results of the DEG analysis (Figure 3A). We performed a KEGG pathways analysis using the DAVID web software. The results of the DAVID analysis revealed significant differential upregulation of the steroidogenesis pathway depending on the sample’s status and clinical outcome. In particular, ovarian steroidogenesis-related genes were significantly differentially regulated according to age and pregnancy outcomes. Therefore, we next focused on the signal pathway identity of each gene cluster using a KEGG pathway analysis. The KEGG pathway analysis results (Figure 3B) revealed that upregulated DEGs in CCs were associated with the ovarian steroidogenesis pathway (LDLR and StAR) and downregulated DEGs were related to pregnancy status (ADCY and 17 beta-hydroxysteroid dehydrogenase 1 (HSD17B1)).

### 3.4. Validation Study of mRNA-seq Data by Real-Time qPCR

**Figure 4:**
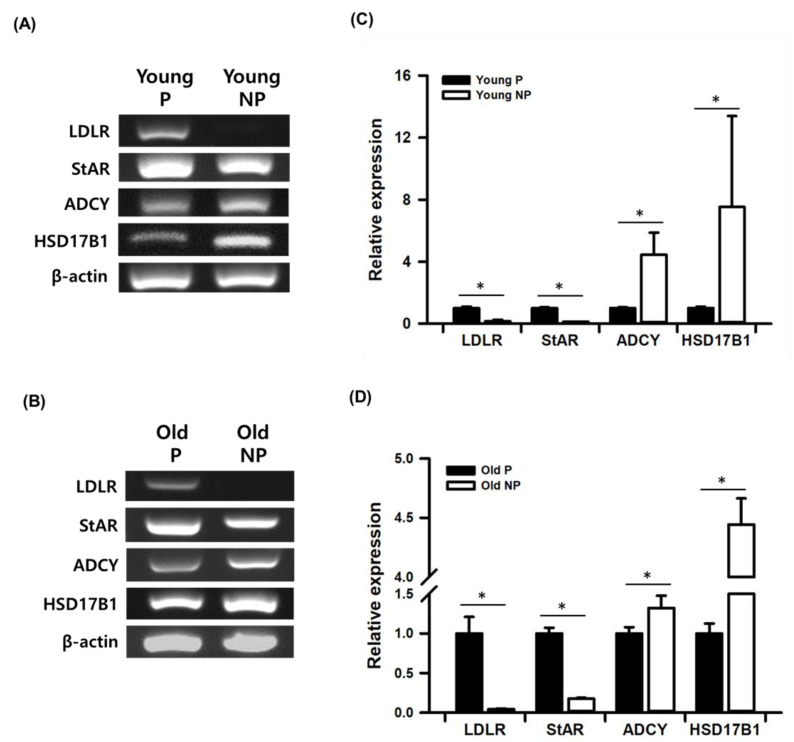
Validation of transcriptome data by RT-PCR. Up- and downregulation of genes were confirmed for LDLR, StAR, ADCY, and HSD17B1; β-Actin was used as a control. Gel images of PCR products are shown for the young (**A**) and old (**B**) pregnancy and non-pregnancy groups. (**C**,**D**) Data of real-time RT-qPCR for up- and downregulated genes, based on NGS data from the pregnancy and non-pregnancy groups. Data are presented as means ± SEM of three replicates. Significant differences are indicated by asterisks (* *p* < 0.05).

Next, we performed real-time qPCR to validate the mRNA-seq data. The samples used for NGS were subjected to RT-PCR with primers specific for up- and downregulated genes. Figure 4A,B show the RT-PCR bands of LDLR, StAR, ADCY, and HSD17B1. The pregnancy group expressed LDLR and StAR at significantly higher levels than the non-pregnancy group but expressed ADCY and HSD17B1 at significantly lower levels. Validation was confirmed by conventional PCR followed by gel electrophoresis and real-time qPCR. Both approaches yielded similar patterns of up- and downregulated genes. The comparisons of the mean relative mRNA levels between pregnancy and non-pregnancy are shown in Figure 4C,D. Therefore, the real-time qPCR data showed that the expressions of the genes ADCY and HSD17B1 were 4-fold and 7-fold higher, respectively, in the young pregnancy group than in the young non-pregnancy group.

## 4. Discussion

In this study, we found specific factors in CCs that were associated with successful pregnancy in advanced aged IVF women using whole-transcriptome NGS analysis. Except for embryo quality and blastocyst rates, the clinical criteria (basal FSH level, AMH, maturation rate, etc.) did not differ significantly between the age and pregnancy groups. The pregnancy group had significantly higher blastocyst quality than the non-pregnancy group. Hierarchical clustering and multidimensional scaling analysis of whole-transcriptome NGS data revealed different up- and downregulated DEG clusters depending on young and age groups with archive pregnancy; upregulated genes included LDLR and StAR, and downregulated genes included ADCY and HSD17B, whose expression is associated with the ovarian steroidogenesis signaling pathway. In addition, based on the validated data, the whole-transcriptome NGS data showed a similar up- and downregulation of gene expression profiles between the young and old pregnancy groups.

Based on the DEG data from whole-transcriptome NGS, the pregnancy group (both young and old) exhibited an upregulation of genes including ZNF117, ITGA2, and SFRP4, which are involved in the functional regulation of GCs, oogenesis, and follicular development in the ovary [27,28,29,30]. In particular, SNORD150, which is encoded in the introns of protein-coding or non-coding genes, plays a role in the modification, maturation, and stabilization of rRNA. SNORD150 has not been shown to play a role in folliculogenesis and ovulation. In the young pregnancy group, the JunD proto-oncogene was upregulated in the ovary. The promoter of the aromatase gene in GCs has been reported to be regulated by activator protein-1 (AP1) during steroidogenesis, but its role in pregnancy has been unclear [31]. Other factors, such as tenascin-C induced interleukin-1 alpha, prostaglandin E2, and prostaglandin F2-alpha, are associated with the proliferation, differentiation, and steroidogenesis in CCs in the ovary and stromal cells in the murine uterus during early pregnancy [32,33,34,35]. In the older age pregnancy group, NME1-NME2 gene expression was 10-fold higher than in the older age non-pregnancy group. To our knowledge, NME1/2 has not been confirmed to play a role in the ovary. The young and old pregnancy groups downregulated genes such as CAMK2N1, DHRS9, PSAT1, and TXNIP, all of which have confirmed functions in the ovary associated with PCOS [36,37,38]. Other downregulated genes did not have identified functions in the mammalian ovary. Both the young and old groups exhibited the downregulation of NGFR, which is involved in early follicular development but has no confirmed role in the late follicular phase. Other downregulated genes did not have reported ovarian functions but had been studied in the context of ovarian cancer. DEG data showed that several genes were associated with age and pregnancy.

DEG data were analyzed using the DAVID functional annotation clustering tool. DEG data used to visualize the KEGG pathway result revealed the up- and downregulation of gene signaling pathways. KEGG pathway mapping of gene set enrichment analysis data revealed that ovarian steroidogenesis was strongly associated with successful clinical pregnancy in patients of advanced age. The presence of LDL is required for maximal progesterone secretion by cultured human granulosa cells [39]. The NGS data revealed the upregulation of factors such as LDLR and StAR in the pregnancy group, which have been reported to play a role in the initiation of steroidogenesis in the ovary [40,41,42]. The major role of LDLR is to serve as the initial importer of precursors for steroids such as E2 and P4, which are involved in gonadotropin-dependent oogenesis, ovulation, and implantation during the luteal phase. However, a pregnancy-related role for LDLR has not been identified in the human IVF cycle. We suggest that LDLR and StAR are the key upregulated factors responsible for successful pregnancy in the old and young groups. StAR is involved in the intracellular mitochondrial cholesterol transfer from cytoplasm to mitochondria for steroidogenesis [43]. Therefore, LDLR and StAR are also key factors for successful pregnancy in the context of steroid metabolism. Even older patients had highly activated steroidogenesis, reflected by the upregulation of LDLR and StAR. Those factors improve embryo quality and blastocyst ratios and help to achieve a successful clinical outcome. We demonstrated that ADCY and HSD17B1 are among the main downregulated factors in the CCs associated with achieving pregnancy in young and old subjects. The expression of ADCY, a downregulated gene, is inhibited in oocyte meiotic prophase I by cyclic adenosine monophosphate (cAMP) [44,45]. Another downregulated gene, HSD17B1, plays a role in testosterone biosynthesis in the human ovary. Consequently, HSD17B1 is associated with hyperandrogenic anovulation, as in PCOS [46,47]. Therefore, the downregulation of HSD17B1 is correlated with the inhibition of androgen hormone synthesis during normal follicular development and ovulation.

This study identified LDLR-linked steroidogenesis activity in cumulus cells as a potentially important factor for reducing infertility in women of advanced age. Infertility is a global health issue, and it is considered that about one in seven couples may have difficulty conceiving. In the clinical field, the number of women of advanced age continues to increase, and accordingly, the successful pregnancy rate through IVF procedure is declining. Globally, the average age at which people marry has increased, and the mean age at which women experience infertility has also dramatically increased over the last decade. Older infertile women have an unmet need to rid themselves of the age-related issues that prevent successful pregnancies and to experience a healthy birthing experience. This study opens the prospect of the development of a new ovarian stimulation protocol that overcomes the issues that prevent pregnancy at an advanced age.

## 5. Conclusions

We identified a specific transcriptome profile in the CCs of young and old IVF women. Up-regulation of LDLR and StAR was associated with embryo quality and clinical pregnancy in infertile women. Those data could be applied to new COS protocols to activate LDLR and StAR in infertile women of advanced age, which may help to increase maturation ratios and pregnancy rates in the IVF cycles. Further studies of LDLR upregulation should be performed in infertile women who have not previously had a successful pregnancy in a large size prospective model.

## Figures and Tables

**Table 1:** Patients’ characteristics and oocyte profiling of women in the young and old groups, with or without pregnancy. BL: blastocyst, CL: cleavage stage embryo. Significant differences are indicated by asterisks (* *p* < 0.05).

	Young Preg (*n* = 8)	Young Non-Preg (*n* = 8)	Old Preg (*n* = 8)	Old Non-Preg (*n* = 8)
Age (years)	31.16 ± 1.16	33.8 ± 1.78	39.83 ± 0.75	40.83 ± 1.83
Serum AMH (ng/mL)	4.49 ± 2.79	2.80 ± 1.27	1.95 ± 1.01	1.99 ± 0.97
BMI (kg/m^2^)	23.30 ± 3.41	21.49 ± 2.56	24.20 ± 3.15	22.33 ± 2.56
Basal FSH (mIU/mL)	7.96 ± 2.99	8.36 ± 2.20	8.23 ± 2.16	7.89 ± 1.47
No. of previous IVF cycles	2.6 ± 1.67	2.0 ± 1.22	1.0 ± 0.81	2.16 ± 1.60
Total Gonadotropin dose	3030 ± 497	2308 ± 820	2525 ± 318	2995 ± 359
No. of antral follicle count	11.25 ± 5.31	8.88 ± 7.64	8.80 ± 3.63	9.33 ± 4.96
No. of retrieved oocytes	13.16 ± 6.43	10.4 ± 2.30	8.66 ± 5.88	10.16 ± 4.99
Maturation rate (%)	77.01 ± 15.31 *	64.77 ± 7.08	87.12 ± 16.11 *	81.22 ± 17.78
No. of MII oocytes	9.5 ± 3.93	6.8 ± 2.04	5.0 ± 3.37	6.66 ± 4.84
Fertilization rate (%)	91.05 ± 8.61	68.85 ± 32.23	83.33 ± 23.57	75.82 ± 17.93
No. of transferred embryo	1.16 ± 0.40	1.63 ± 0.92	2.33 ± 0.81	2.00 ± 0.00
Stage of transferred embryo				
BL (%)/CL (%)	100 */0	62.5/37.5	83.33 */16.67	66.66/33.33

Hierarchical clustering and MDS (multidimensional scaling) of transcriptome profiling in the CCs.

## Supplementary Materials

The following are available online at https://www.mdpi.com/article/10.3390/cells10092389/s1, Table S1: (A) both young and old pregnancy group up- and downregulation top 10 gene list, (B) young or old group only upregulation gene top 10 lists, (C) Young or old group only downregulation gene top 10 lists. Table S2 Primer sequences used for real-time qPCR.
