# Supplementary material for: Upregulation of Low-Density Lipoprotein Receptor of the Steroidogenesis Pathway in the Cumulus Cells Is Associated with the Maturation of Oocytes and Achievement of Pregnancy
Source: Cells. 2021 Sep 11;10(9):2389. doi: 10.3390/cells10092389 (PMC8465166; doi:10.3390/cells10092389)
Supplement: Supplementary file 1 [file cells-10-02389-s001.zip › cells-1347130-supplementary.pdf]

Table S1

(A) Both Young and Old pregnancy group up- and down-regulation top 10 gene list

	Gene Symbol	Description	Young : Non-Pregnancy vs Pregnancy Fold Change	Old : Non-Pregnanc y vs Pregnancy Fold Change
Up regulation	SNORD159	small nucleolar RNA, C/D box 159	2.018469	2.160406
	ZNF117	zinc finger protein 117	3.904157	2.635705
	ITGA2	integrin subunit alpha 2	2.414954	3.019805
	LOC102723753	HECT and RLD domain containing E3 ubiq uitin protein ligase 2 pseudogene	2.824969	3.533761
	SFRP4	secreted frizzled related protein 4	2.649269	3.617478
	NME1-NME2	NME1-NME2 readthrough	3.532060	17.918974
Down regulation	NGFR	nerve growth factor receptor	-13.181860	-7.632480
	ADNP	activity dependent neuroprotector home-obo x	-6.182802	-3.508153
	SLC44A2	solute carrier family 44 member 2	-5.652775	-4.948898
	LOC728715	ovostatin homolog 2	-5.646341	-2.095229
	HMGB2	high mobility group box 2	-4.669963	-3.403419
	PLCD1	phospholipase C delta 1	-4.559903	-4.480157
	SDSL	serine dehydratase like	-4.485008	-2.728038
	ACTA1	actin, alpha 1, skeletal muscle	-3.889844	-2.643790
	DUSP15	dual specificity phosphatase 15	-3.829421	-2.535768
	ZMIZ1-AS1	ZMIZ1 antisense RNA 1	-3.759088	-2.906539

Table S1 continuous

(B) Young or old group only up regulation gene top 10 lists

	Gene Symbol	Description	Non-Pregnancy vs Pregnancy Fold Change
Young Up regulation	JUND	JunD proto-oncogene, AP-1 transcription f actor subunit	11.348648
	MIR6506	microRNA 6506	8.990142
	SNORD116-4	small nucleolar RNA, C/D box 116-4	7.544863
	MIR4444-1	microRNA 4444-1	5.905006
	EIF3CL	eukaryotic translation initiation factor 3 su bunit C like	5.225406
	RGS4	regulator of G protein signaling 4	4.588431
	LOC102724238	uncharacterized LOC102724238	4.066461
	TNC	tenascin C	4.001524
	ZNF117	zinc finger protein 117	3.904157
	NME1-NME2	NME1-NME2 readthrough	3.532060
Old Up regulation	NME1-NME2	NME1-NME2 readthrough	17.918974
	SCARNA28	small Cajal body-specific RNA 28	7.508940
	SNORD116-6	small nucleolar RNA, C/D box 116-6	5.282785
	C1GALT1C1	C1GALT1 specific chaperone 1	4.824189
	PWARSN	Prader Willi/Angelman region RNA, SNRP N neighbor	4.686805
	CTSK	cathepsin K	4.325185
	HBB	hemoglobin subunit beta	3.954614

SLX1A	SLX1 homolog A, structure-specific en-don uclease subunit	3.895302
GLUL	glutamate-ammonia ligase	3.720067
KLF6	Kruppel like factor 6	3.715995

Table S1 continuous

(C) Young or old group only down regulation gene top 10 lists

	Gene Symbol	Description	Non-Pregnancy vs Pregnancy Fold Change
Young down regulation	SNORA18	small nucleolar RNA, H/ACA box 18	-20.929513
	SCARNA28	small Cajal body-specific RNA 28	-19.033759
	NGFR	nerve growth factor receptor	-13.181860
	CBLN1	cerebellin 1 precursor	-7.511419
	CAMK2N1	calcium/calmodulin dependent protein kinase II inhibitor 1	-6.323503
	ADNP	activity dependent neuroprotector homeobox	-6.182802
	LUZP6	leucine zipper protein 6	-5.970195
	MIR4444-2	microRNA 4444-2	-5.857645
	DHRS9	dehydrogenase/reductase 9	-5.832381
	SLC44A2	solute carrier family 44 member 2	-5.652775
Old down Regulation	PSAT1	phosphoserine aminotransferase 1	-19.425404
	HLA-DRB5	major histocompatibility complex, class II, DR b eta 5	-13.534710

TXNIP	thioredoxin interacting protein	-10.713780
TRIB3	tribbles pseudokinase 3	-7.701359
NGFR	nerve growth factor receptor	-7.632480
RGS4	regulator of G protein signaling 4	-7.545754
SNORD116-4	small nucleolar RNA, C/D box 116-4	-7.379899
PCK2	phosphoenolpyruvate carboxykinase 2, mito-cho ndrial	-7.325029
VTRNA1-3	vault RNA 1-3	-6.196869
VSTM2L	V-set and transmembrane domain containing 2 like	-6.065403

Table S2 Primer sequences used for real-time qPCR

Gene		Sequence (5`-3`)
LDLR	F	TGC TGG CAG AGG AAA TGA GA
	R	CTG GTT GTG GCA AAT GTG GA
StAR	F	AGC ATC ATC AAC CAG GTC CT
	R	TAA ACA CGA ACC CCA CCC AT
ADCY	F	CAA CGT GGC ATC CTG AAG AG
	R	GAA CGA TCT GGG CAC ACA TC
HSD17B1	F	ATG GGG CTG CCT TTC AAT GA
	R	GAT CAG GCT CAA GTG GAC CC
β-actin	F	ACA ATG TGG CCG AGG ACT TT
	R	TGT GTG GAC TTG GGA GAG GA
